# Performance of Deep-Learning Solutions on Lung Nodule Malignancy Classification: A Systematic Review

**DOI:** 10.3390/life13091911

**Published:** 2023-09-14

**Authors:** Hailun Liang, Meili Hu, Yuxin Ma, Lei Yang, Jie Chen, Liwei Lou, Chen Chen, Yuan Xiao

**Affiliations:** 1 School of Public Administration and Policy, Renmin University of China, Beijing 100872, China; hliang@ruc.edu.cn (H.L.);; 2 Department of Gynecology, Baoding Maternal and Child Health Care Hospital, Baoding 071000, China; humeili001@126.com; 3 Key Laboratory of Carcinogenesis and Translational Research (Ministry of Education/Beijing), Beijing Office for Cancer Prevention and Control, Peking University Cancer Hospital & Institute, Beijing 100142, China; 4 School of Statistics, Renmin University of China, Beijing 100872, China; 5 Blockchain Research Institute, Renmin University of China, Beijing 100872, China

**Keywords:** lung nodules, deep learning, convolutional neural network, low-dose CT

## Abstract

Objective: For several years, computer technology has been utilized to diagnose lung nodules. When compared to traditional machine learning methods for image processing, deep-learning methods can improve the accuracy of lung nodule diagnosis by avoiding the laborious pre-processing step of the picture (extraction of fake features, etc.). Our goal is to investigate how well deep-learning approaches classify lung nodule malignancy. Method: We evaluated the performance of deep-learning methods on lung nodule malignancy classification via a systematic literature search. We conducted searches for appropriate articles in the PubMed and ISI Web of Science databases and chose those that employed deep learning to classify or predict lung nodule malignancy for our investigation. The figures were plotted, and the data were extracted using SAS version 9.4 and Microsoft Excel 2010, respectively. Results: Sixteen studies that met the criteria were included in this study. The articles classified or predicted pulmonary nodule malignancy using classification and summarization, using convolutional neural network (CNN), autoencoder (AE), and deep belief network (DBN). The AUC of deep-learning models is typically greater than 90% in articles. It demonstrated that deep learning performed well in the diagnosis and forecasting of lung nodules. Conclusion: It is a thorough analysis of the most recent advancements in lung nodule deep-learning technologies. The advancement of image processing techniques, traditional machine learning techniques, deep-learning techniques, and other techniques have all been applied to the technology for pulmonary nodule diagnosis. Although the deep-learning model has demonstrated distinct advantages in the detection of pulmonary nodules, it also carries significant drawbacks that warrant additional research.

## 1. Background

Lung cancer is the most commonly diagnosed cancer and a leading cause of cancer mortality worldwide, accounting for 11.6% of total new cases of cancer and 18.4% of total cancer deaths [1]. Despite significant advances in lung cancer diagnosis and treatment, many cases continue to be diagnosed at a late stage, which carries a dismal prognosis: a five-year relative survival of 28.6% for cases with regional spread and 4.2% for those with distant metastasis [2]. The detection of lung cancer at an early and treatable stage is a key factor for improved outcomes and can be realized through screening. Lung cancer screening with low-dose computed tomography (LDCT) has been demonstrated to be effective in reducing lung cancer mortality by 20% compared to chest radiography [3] and has been recommended to high-risk populations for annual screening [4,5].

The widespread use of CT examinations, however, generates a large amount of image data that needs to be reviewed and interpreted by radiologists, considerably increasing their workload. The current assessment of pulmonary nodule changes mainly relies on visual comparisons and diameter measurements based on baseline and follow-up axial images. Additionally, a significant number of ambiguous pulmonary nodules, of which more than 95% are non-cancerous, are found during lung cancer screening [3]. The necessary follow-up diagnostic evaluations provide a major burden to the healthcare system and, at the same time, raise possibilities for machine learning solutions to enhance nodule detection and malignancy classification.

Long-term research on computerized systems for lung lesion detection has focused on recognizing nodules reliably, especially tiny nodules, and lowering false-positive rates [6]. Finding the nodule was once the primary concern of many researchers, but recently, that focus has shifted to determining the nodule’s malignancy status. From a technical standpoint, computer-aided diagnosis has progressed from traditional machine learning approaches, which mostly include manually generated feature extraction and feature categorization, to deep learning. In comparison to traditional approaches, deep learning has a greater ability to represent varied lung nodule shapes, margins, and compositions. This is carried out by allowing the machine to learn from limited annotated LDCT data and automatically derive features. Deep learning may hold promise in improving the classification of these nodules and predicting malignancy in the era of population-based lung cancer screening. How to effectively manage a large number of screen-detected nodules, especially indeterminate pulmonary nodules (IPD), needs to be carefully addressed. Therefore, in this study, we aimed to summarize the performance of existing deep-learning solutions on lung nodule malignancy classification in LDCT.

## 2. Methods

### 2.1. Data Sources and Search Strategy

A systematic literature review was performed to identify studies that evaluated the performance of deep-learning solutions on lung nodule malignancy classification. Databases of PubMed and ISI Web of Science were searched for eligible articles from inception to 31 December 2021. The search phrase utilized was an LDCT, AI, and lung nodule combination, as described in Appendix A. Additional pertinent studies were checked for in the bibliographies of qualified papers. The PRISMA recommendations were followed in conducting and reporting this review [7].

### 2.2. Study Selection

Published studies were eligible for inclusion if they reported the indicators reflecting the performance of a deep-learning system in classifying lung nodules as malignant or benign on LDCT in the general population. This review was restricted to original articles published in English. We excluded studies if they did not address lung nodules or cancer, were not related to the diagnosis or screening of lung nodules, did not use artificial intelligence, or were not based on LDCT. Studies that only introduced the technical methods used in the artificial intelligence system or the performance was merely evaluated in a phantom study were also excluded. Additionally, studies in which the goal was nodule identification rather than cancer classification or in which deep learning was used to perform cancer classification were eliminated. Studies that described their objective as “cancer diagnosis”, “malignancy prediction”, or other similar terms were reviewed as long as they were able to determine the status of malignancy. We included articles from the same research group, if they were found, that used various technological approaches.

### 2.3. Data Extraction and Synthesis

Two investigators (HL and LY) independently extracted data from the included studies into a standardized form. First, we gathered and summarized data on each article’s author, publication year, and country of origin. Secondly, the deep-learning system’s properties; and thirdly, the dataset’s characteristics, including the cohort’s name and location, the number of patients with benign and malignant nodules, and the size of the nodules. Area under the curve (AUC), accuracy, sensitivity, and specificity are the final performance metrics. The investigators discussed and reviewed the data again in order to settle any discrepancies.

As this review focuses on the differences in system/algorithm used and the corresponding performance, no specific scoring system was developed to rate the quality of the articles included. Instead, we presented and summarized the detailed information about the system/algorithm and the dataset the performance was tested on.

Microsoft Excel 2010 (Microsoft Corporation, Albuquerque, NM, USA) and SAS version 9.4 (SAS Institute Inc., Cary, NC, USA) were used for data extraction and to plot the figures.

## 3. Results

### 3.1. Literature Search Results

**Figure 1:**
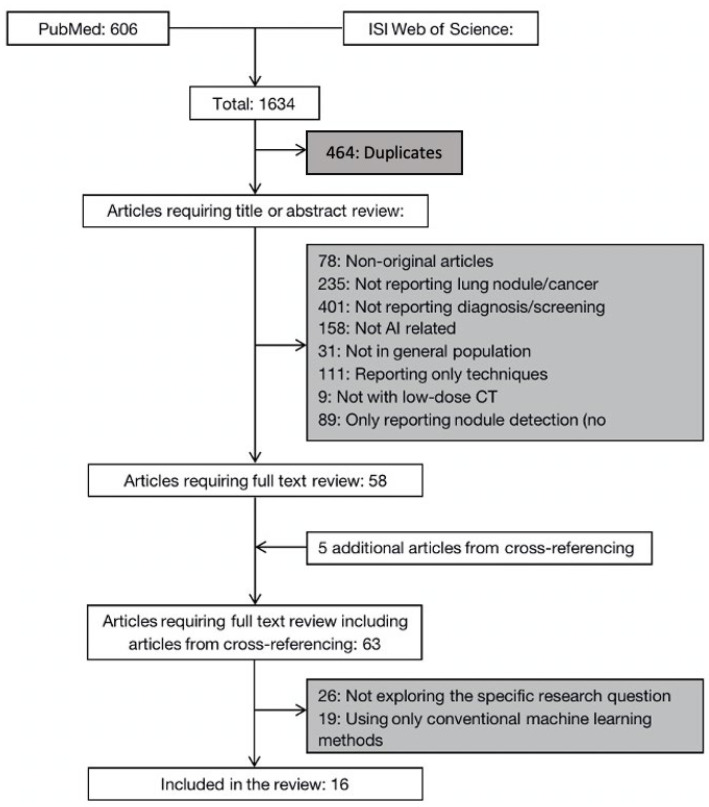
Flow diagram of systematic literature search. AI = artificial intelligence; CT = computed tomography.

The initial search yielded a total of 1634 articles, 606 from PubMed and 1028 from Web of Science. After removing the duplicates and scanning the title and abstract, 58 articles and five additional articles identified from cross-referencing were selected for a full-text review. Of these, 16 studies met the selection criteria and were included in our analysis. The study selection process is summarized in Figure 1.

### 3.2. Deep-Learning Solutions on Lung Nodule Malignancy Classification

The application of a CAD system in the early diagnosis of lung cancer usually includes the following steps: data preprocessing, lung region segmentation, candidate nodule detection and segmentation, and nodule diagnosis [8]. Our study was mainly concerned with the last step that the performance of deep-learning solutions on lung nodule malignancy classification. Machine learning from end to end is known as deep learning. This model does a one-step nodule detection and direct image processing. Deep-learning-based CAD systems can successfully address key issues in the early diagnosis of lung cancer, such as feature extraction, lung nodule recognition, and the decrease in false-positive rates [9]. Deep-learning models are historically divided into supervised learning and unsupervised learning, in which supervised learning needs to use data with classification labels. Supervised learning models include convolutional neural networks (CNNs) and mass-training artificial neural networks (MTANNs). Unsupervised learning includes an automatic encoder (AE) and deep belief network (DBN). The literature we screened included the above two model types, and the details are included in Table 1.

### 3.3. Convolutional Neural Network (CNN)

Convolutional neural network (CNN) is the most widely used deep-learning model in the field of medical imaging and is composed of the input layer, convolution layer, activation function, pooling layer, full connection layer, and output layer. Machine learning from end to end is known as deep learning. This model does a one-step nodule detection and direct image processing. Deep-learning-based CAD systems can successfully address key issues in the early diagnosis of lung cancer, such as feature extraction, lung nodule recognition, and the decrease in false-positive rates.

CNN used in the detection and classification of lung nodules mainly includes CNN, deep CNN, multi-view CNN, multi-crop CNN, multi-level CNN, and so on. Some classical nodule classification algorithms have been enhanced via deep learning. Using each feature type, Xie et al. trained an AdaBoosted back propagation neural network (BPNN) and fused the conclusions reached via three classifiers. The algorithm used a deep convolutional neural network (DCNN) to automatically learn the feature representation of nodules on a slice-by-slice basis, a Fourier shape descriptor to describe the heterogeneity of nodules, and a gray level co-occurrence matrix (GLCM)-based texture descriptor to describe the texture. This method combines deep CNN feature learning with backpropagation neural networks.

The performance of the Fuse-TSD algorithm was assessed using the area under the receiver operator curve (AUC). The algorithm achieved an AUC of 96.65%, 94.45%, and 81.24%, respectively, higher than the AUC obtained using the LeNet-5 feature, GLCM-based texture descriptor, and Fourier shape descriptor, respectively [21].

Some studies explored two-dimensional (2D) CNN for the categorization of lung nodules with the use of deep learning. Shen et al. used a multi-crop CNN to solve the lung nodule malignancy classification problem for CT images. They used the LIDC-IDRI database, which had 1243 indeterminate nodules in addition to 880 benign nodules and 495 malignant nodules. The multi-crop CNN extracted multi-scale features by employing a multi-crop pooling strategy. Convolutional features obtained from the original image or pooled features served as the inputs for the multi-crop pooling technique. Then, to extract the information about the nodules, the study repeatedly applied max-pooling to the multi-scale characteristics. The accuracy of multi-crop CNN was 87.14%, and the AUC was 0.93 [22].

Liu et al. proposed a multi-view CNN for classifying nodule types in CT images. Unlike traditional CNNs, an MV-CNN takes multiple views of each entered nodule. Experiments showed that the MV-CNN achieved an AUC of 0.981, sensitivity of 0.9049, and specificity of 0.9991 [20]. Paul et al. developed a hybrid model for lung nodule malignancy prediction utilizing convolutional neural network ensembles. This study divided nodules into large and small nodules based on different clinical guideline thresholds. CNNs were designed and trained over each of these groups individually. The size of solid nodules was used to split the database into three groups of 6 and 8 mm. This study also analyzed clinical features, such as gender, family history of lung cancer, and smoking history [17].

The multi-crop pooling technique used convolutional features extracted from the original image or pooled features as inputs. The team then repeatedly performed max-pooling to the multi-scale features to retrieve the information about the nodules.

Lyu et al. proposed a multi-level convolutional neural network (ML-CNN) to investigate the problem of lung nodule malignancy classification. Three CNNs were in ML-CNN models to extract multi-scale features in lung nodule CT images. This study further flattened the output of the last pooling layer into a one-dimensional vector for every level and then concatenated them. The methodology assisted in improving model performance. According to experimental findings, ML-CNN attained 84.81% accuracy without the use of any additional manual preprocessing algorithms [25].

Nishio et al. used a deep convolutional neural network (DCNN) for CADx of the ternary classification. The conventional CAD extracted features using a local binary pattern and then fed the features to SVM for classification tasks. The deep CNN was modified via VGG-16. ImageNet was used for transfer training. The validation accuracy of CNN with transfer learning achieved 68.0% better than CNN without transfer learning and conventional CAD [10]. Three-dimensional (3D) CNN was examined in further literature. The network depth of 3D CNN is greater than that of 2D CNN. It can extract a number of different features from the spatial information of pulmonary nodules in CT images, which significantly increases the recognition accuracy. When categorizing the same data set and using the same network parameter settings, 3D CNN is more accurate than 2D CNN.

Ardila et al. propose a deep-learning algorithm that uses a patient’s current and prior computed tomography volumes to predict the risk of lung cancer. First, the study built a three-dimensional (3D) CNN model that analyzes whole-CT volumes, end to end.

Second, they trained a CNN region-of-interest (ROI) identification model (the “cancer ROI detection model”) to find 3D cancer candidate regions in the CT volume. To train this model, more bounding box labels were gathered. Last but not least, the study created a CNN cancer risk prediction model that uses outputs from both the full-volume model and the cancer ROI detection model. The model’s AUC for predicting lung cancer was 94.4% in a year [11].

Li et al. proposed an algorithm fusing the features achieved from handcrafted features (HF) and deep convolutional neural network (DCNN) for predicting lung nodule malignancy. The study initially extracted twenty-nine handcrafted features based on a grey-level cooccurrence matrix (GLCM) averaged from five grey levels, four distances, and thirteen directions. Then, they trained 3D CNNs to extract the CNN features learned at the output layer. There are three 3D CNNs in total, modified from 2D CNNs, namely AlexNet, VGG-16 Net, and Multi-crop Net. For each 3D CNN, the CNN features combined with the 29 handcrafted features were used as the input for the support vector machine (SVM) coupled with the sequential forward feature selection (SFS) method to select the optimal feature subset and construct the classifiers. The fusing algorithm achieved an AUC of 0.9303, an accuracy of 88.58%, a sensitivity of 82.60%, and a specificity of 91.82% [26].

In the Ozdemir et al. study, an attention-based multiple instance learning (MIL) framework was used to train their malignancy classification network. The MIL framework is based on a convolutional neural network shared by all selected candidates, followed by a combination layer that combines the features of each candidate using an attention mechanism. The model finally achieved an AUC of 0.87 [16].

Zhao et al. created a CNN model that fuses multi-scale feature fusion with multi-attribute grading to classify lung nodules as benign or malignant. Building a multi-task network (MSMT), which for the first time coupled multi-scale features with multi-attribute classification, was the initial stage. This network was then used to classify benign and malignant lung nodules. The experimental results showed the AUCs of the model were 0.979, 93.92%, 92.60%, and 96.25%, respectively [18]. Additionally, Xie et al. proposed the use of restricted chest CT data to distinguish between benign and malignant nodules using a multi-view knowledge-based collaborative (MV-KBC) deep model.

By splitting a 3D lung nodule into nine fixed images, the model was able to understand the features of 3D lung nodules. The study built a knowledge-based collaborative (KBC) submodel for each view, with three different types of image patches intended to fine-tune three pre-trained ResNet-50 networks that, respectively, represent the nodules’ overall appearance, voxel heterogeneity, and shape heterogeneity.

Xie et al. used the nine KBC submodels to classify lung nodules with an adaptive weighting scheme learned during the error back propagation, which enables the MV-KBC model to be trained in an end-to-end manner. The penalty loss function was used for a better reduction in the false negative rate with a minimal effect on the overall performance of the MV-KBC model. The results showed that the MV-KBC model achieved an accuracy of 91.60% for lung nodule classification with an AUC of 95.70% [23].

Huang et al. constructed a deep-learning algorithm (referred to as DeepLR) from 25,097 participants in a National Lung Screening Trial, and the algorithm was proved in double-blinded trials. The model achieved AUC of 0.968, 0.946, and 0.899, respectively, indicating the accuracy of DeepLR scores to predict lung cancer incidence at 1 year, 2 years, and 3 years. [12]. Asuntha et al.’s study used a novel FPSOCNN for lung cancer classification and considered it to reduce the computational complexity of CNN. The study also compared FPSOCNN with other methodologies, and the final results showed that FPSOCNN outperformed them all. The model had 94.97% accuracy, 96.68% sensitivity, and 95.98% specificity, respectively [14]. Lei et al.’s study first developed a soft activation mapping (SAM) to enable fine-grained lung nodule shape and margin (LNSM) feature analysis with a CNN so that it can access rich discrete features. They then further proposed a high-level feature enhancement scheme (HESAM) to localize LNSM features by combining high-level convolutional features with SAM. The method achieved an accuracy of 99.13%, a sensitivity of 0.9705, and a specificity of 0.9921 [15]. Tajbakhsh et al. compare the performance of massive-training artificial neural networks (MTANNs) and CNNs for distinction in CT images, showing that MTANNs with limited training data outperform CNNs in the experiment. MTANNs achieved an AUC of 0.8806, which was greater than the CNN model with an AUC of 0.7755 [27].

### 3.4. Autoencoder (AE)

AE is an unsupervised deep-learning model that primarily consists of input, hidden, and output layers. The encoder and decoder were hidden layers. The coding process occurs from the input layer to the hidden layer in AE, whereas the decoding process occurs from the hidden layer to the output layer. In comparison to the conventional manual tag extraction, feature extraction via coding and decoding is more objective and trustworthy. At present, the AE used in the CAD system of pulmonary nodules include stack AE [28], denoising autoencoder (DAE), and stack DAE. A study by Sun et al. used a stacked denoising autoencoder (SDAE) to extract parameters, and then applied the parameters to a supervised neural network, with an AUC of 0.852 ± 0.025 higher than using CNN [24].

### 3.5. Deep Belief Network (DBN)

The concept of DBN was proposed by Hinton and Salakhutdinov [29] in 2006, which is defined as a probability generation model with multi-layer neurons. The basic structure of DBN is a Restricted Boltzmann machine, which is characterized by a full connection between the visible layer and hidden layer but no intra-layer connection between the hidden layer and visible layer [30]. The connection between the top two layers is undirected and forms associative memory. All connections between layers point in the direction of the layer that is closest to the data. Each neuron in the bottom layer represents a certain dimension of the data vectors, which are represented by the bottom layer.

This connection mode is the basis of its efficiency. Hua et al. proposed a deep belief network (DBN) for malignant and benign classification in CT images. The DBN was established by constructing stacked RBMs iteratively with three hidden layers and a visible layer. Tested on the LIDC data set, DBN (sensitivity of 73.4% with specificity of 82.2%) and deep CNN (sensitivity of 73.3% with specificity of 78.7%) outperformed k-nearest neighbors with SIFT and LBP (sensitivity of 75.6 with specificity of 66.8%), and support vector machine with fractal analysis (sensitivity of 50.2% with specificity of 57.2%) [19]. In addition, the latest proposed adaptive squeeze-and-shrink (ASAS) denoising technique optimizes the precision by 18.03% and sensitivity by 7.64% [31].

## 4. Discussion

With increasing lung tissue sample size and diversity, the Lung Image Database Consortium and Image Database Resource Initiative, LIDC-IDRI, and other databases continuously provide a large number of expert-labeled lung CT image data. These factors address the necessary conditions for the development of CT technology [32]. The examination of lung nodules and the evaluation of malignant tumors have benefited significantly from CT technology as a result of the development of computer technology [33]. The image processing approach, traditional machine learning method, and deep-learning method of artificial intelligence have all been developed for use in lung nodule detection [34]. Deep learning is now considered the brand-new method in medical image analysis, and it has been shown to outperform traditional machine learning methods in several domains with more precise results and better generalizability. But, the limitations of deep learning may include more data required for training and potential robustness behavior compared with traditional methods [35]. Due to the experienced performance of CT in accurately identifying malignant pulmonary nodules, which is crucial to the diagnosis of lung cancer, patients’ chances of cure are apparently improved. The wide application of deep-learning methods in computer vision also makes CT play an increasingly prominent role in the detection of malignant nodules [36].

Through a systematic review, we found that the convolutional neural network (CNN) has been extensively applied in the diagnosis of pulmonary nodules. Numerous studies have shown that the use of deep-learning technology in a variety of fields, including the categorization of lung nodules and the end-to-end detection of lung cancer, is adequate to improve the AUC, accuracy, sensitivity, and specificity of effects performance. The spatial three-bit information of pulmonary nodules can be used to anticipate using deep-learning technology as a way of multi-level feature learning. CNN has an outstanding advantage in diagnosis sensitivity and accuracy compared with the traditional computer-aided detection system (CAD), and its false positive is also controlled [13]. According to the study, discriminative results may be obtained by integrating deep belief networks and convolutional neural network models into the standard CAD image analysis pipeline under the condition of executing the nodule classification of tomographic images [37].

Deep-learning approaches use multi-layer neural networks to process medical data, increasing the predictive power of several specific applications in different clinical domains. In addition, the deep-learning algorithm outperforms other approaches in terms of accuracy, computational efficiency, and extensibility [38]. A deep architecture also has the capacity to integrate many data sets into heterogeneous data types and offer more generalization because of its hierarchical learning structure [39]. An additional study has claimed that combining Inception-V3 and MobileNet classifiers with semantic segmentation and transfer learning is capable of improving significantly the performance of deep-learning models in classifying 3D lung CT scan images [40].

## 5. Conclusions

In conclusion, this paper discussed the detection and classification methods of lung nodules based on a deep-learning model architecture. Currently, a number of studies have applied cutting-edge methods of deep-learning algorithms to lung nodule detection and classification. The efficacy of the models has recently been impacted by various network architectures. It is difficult to assess directly the superiority of model performance because the lung CT image databases and datasets used by different researchers frequently vary. For instance, for CNN networks, the more complex the overall model architecture, the better results achieved in natural image classification recognition and possibly the better nodule classification. Despite the great achievements made in the field of lung nodule detection and classification, it is undeniable that there is still a wide range of research content, such as unsupervised learning algorithms and CAD systems, requiring in-depth exploration in the future. However, the following two issues should be addressed in the future. First, a common limiting factor in most studies is the scarce training data, which negatively affects the robustness and effectiveness of the model. Second, since most previous studies were retrospective analyses, it is essential to conduct more prospective large-sample analyses and rigorous real-world clinical practice in heterogeneous settings in order to verify their real-world practicality.

## Figures and Tables

**Table 1:** Classification or prediction techniques for lung nodules.

Author (Year)	Country	Method (System Structure)	Dimension	Data Set			Effects Performance				Theme
				Name	Location	Size	AUC	Accuracy	Sensitivity	Specificity	Diag. or Predc.?
Nishio (2018) [10]	Japan	**DCNN** Classification: deep CNN modified via VGG-16 CNN	2D	Hospital Patients data	Japan	412 benign nodules 571 primary lung cancers 253 metastatic lung cancers	Image of 56,112,224, the best accuracies were 66.7%, 64.7%, 68.0%.				Lung nodule classification
Ardila (2019) [11]	US	**Deep-learningCNN** Cancer risk prediction: a deep-learning algorithm that uses a patient’s current and prior CT volumes	3D	NLST	US	42,290 CT cases from 14,851 patients, training set (70%), tuning set (15%), test set (15%)	AUC of 94.4% (95% confidence interval, 91.1–97.3) in 1 year				End-to-end lung cancer screening
Huang (2019) [12]	US	**Deep-learningalgorithm** Cancer incidence prediction at 1–3 years: compare accuracy of DeepLR scores and volume doubling time	/	NLST PanCan	US	Training set: 25,097 participants from NLST Validation set:2294 individuals from PanCan	AUC of 0.968 (SD 0.013) with 1-year AUC of 0.946 (SD 0.013) with 2-year AUC of 0.899 (SD 0.017) with 3-year				Prediction of lung cancer risk
Li (2019) [13]	China	**DCNN and handcrafted features** Propose fusion algorithm that combines handcrafted features into the features learned at the output layer of a 3D deep CNN	3D	LIDC IDRI	US	431 malignant nodules 795 benign nodules	AUC of 0.9303, accuracy of 88.58%, sensitivity of 82.60%, specificity of 91.82%				Predicting Nodule Malignancy
Asuntha (2020) [14]	India	**FPSOCNN** Feature extraction:HoG, wavelet transform-based features, LBP, SIFT, Zernike Moment Classification: use 7 methods, the best if FPSOCNN	/	Arthi Scan Hospital Data	India	1000 malignant nodules	Accuracy of 94.97%, Sensitivity of 96.68%, Specificity of 95.89%				Lung cancer classification
Lei (2020) [15]	China	**HESAM:classify nodules through shape and margin** Features extraction: SAM to enable LNSM feature analysis with CNN; HESAM to localize LNSM features.		LIDC IDRI	US	510 malignant nodules 635 benign nodules	Accuracy of 99.13%, Sensitivity of 0.9705, Specificity of 0.9921				Lung nodule classification
Ozdemir (2020) [16]	US	**CNN** Classification: MIL framework to train malignant classification network	3D	LIDC-IDRI Kaggle stage-2 LUNA16	US	LUNA16: 888 patients + 1186 annotated nodules Kaggle stage-2: 153 malignant + 353 benign nodules	AUC of 0.87				Lung cancer Diagnosis
Paul (2020) [17]	US	**CNN ensembles** Malignant prediction made a hybrid model using an ensemble with CNN models of clinical and size information to enhance malignancy prediction.	2D	COCO NLST	US	82 malignant nodules 152 benign nodules	AUC of 0.9, accuracy of 83.12%				lung nodule malignancy prediction
Zhao (2020) [18]	China	**MSMT** Classification: A multi-stream multi-task network	3D	LIDC-IDRI	US	450 malignant nodules 554 benign nodules	AUC of 0.979, Accuracy of 93.92%, Sensitivity of 92.60%, Specificity				benign and malignant classification of nodules
Hua (2015) [19]	China (Taiwan)	**DBN** Classification: Deep Belief Network and CNN to classify	2D	LIDC	US	2545 nodules from 1010 scans	Malignant nodules: sensitivity of 73.4% specificity of 82.2%				classification of lung nodules
Liu (2017) [20]	China	**MV-CNN** Classification: Multi-view Convolutional Neural Networks, it takes multiple views of each entered nodule	2D	LIDC-IDRI	US	96 patients 3540 malignant nodules 764 benign nodules	AUC of 0.981, Sensitivity of 0.9049, Specificity of 0.9991				Lung Nodule Classification
Xie (2018) [21]	China/Australia	Features Extraction: co-occurrence matrix, Fourier shape descriptor and deep CNN Classification: AdaBoosted back propagation neural network	2D	LIDC-IDRI	US	The first data set contains 1324 benign and 648 malignant; the second contains 2021 benign and 648 malignant; the third contains 1324 benign and 1345 malignant.	AUCs of 0.9665, 0.9445, and 0.8124 for three data sets, Accuracies of 89.53%, 87.74%, 71.93% for three data sets Malignant nodules: Sensitivities of 84.19%, 81.11%, and 59.22% with specificity of 92.02%, 89.67%, and 84.85% for three data sets				Classification of lung nodules
Shen (2017) [22]	China/US	Classification: multi-crop **CNN**	2D, 3D	LIDC-IDRI	US	880 benign nodules 495 malignant nodules 1243 uncertain nodules	AUC of 0.93 Accuracy of 87.14%				Lung nodule malignancy suspiciousness classification
Xie (2019) [23]	China/Australia	**MC-CNN** Classification: Multi-view knowledge-based collaborative (MV-KBC) deep model to separate malignant from benign nodules	3D	LIDC-IDRI	US	644 malignant nodules 1301 benign nodules	AUC of 0.957 Accuracy of 91.76%				Benign-Malignant Lung Nodule Classification
Sun (2017) [24]	China	**DBA and SDAE** Classification: LeCun CNN, deep belief network, and stacked denoising autoencoder (SDAE)	2D	LIDC	US	1018 scans (41,372 benign nodules and 47,576 malignant nodules)	AUC of 0.899 ± 0.018 via CNN 0.852 ± 0.025 via SDAE				Computerized Lung Cancer Diagnosis
Lyu (2018) [25]	China Australia	**ML-CNN** Classification: use multi-level convolutional neural network	2D	LIDC-IDRI	US	1018 cases from 1010 patients	Accuracy 84.81%				Lung Nodules Classification

## Appendix A. Search Strategy

(1) (CT or CAT or (computed and tomography)) OR ((CT or CAT or (computed and tomography)) and (scan or scanning or screen or screening)) OR ((spiral or helix or helical) and CT or CAT or (computed and tomography)) OR (((spiral or helix or helical) and CT or CAT or (computed and tomography)) and (scan or scanning or screen or screening))
(2) (low and dose) or low-dose or LDCT or (ultralow and dose) or ultralow-dose or ULDCT
(3) (artificial and intelligence) or AI or (computer and assisted) or computer-assisted or (neural and network) or (machine and learning) or (deep and learning)
(4) ((lung or pulmonary or bronchial) AND (neoplasm or nodule or lesion or cancers or neoplasms or nodules or lesions or cancer or carcinoma or carcinomas))
(5) 1 and 2 and 3 and 4
(6) Limit 5 to English language
